# Impact of out-of-hours admission on patient mortality: longitudinal analysis in a tertiary acute hospital

**DOI:** 10.1136/bmjqs-2017-006784

**Published:** 2017-09-29

**Authors:** Lu Han, Matt Sutton, Stuart Clough, Richard Warner, Tim Doran

**Affiliations:** 1 Department of Health Sciences, University of York, York, UK; 2 Manchester Centre for Health Economics, University of Manchester, Manchester, UK; 3 Haelo, Salford, UK; 4 Salford Royal Foundation Trust, Salford, UK

**Keywords:** health services research, healthcare quality improvement, patient safety, statistics

## Abstract

**Background:**

Emergency hospital admission on weekends is associated with an increased risk of mortality. Previous studies have been limited to examining single years and assessing day—not time—of admission. We used an enhanced longitudinal data set to estimate the ‘weekend effect’ over time and the effect of night-time admission on all-cause mortality rates.

**Methods:**

We examined 246 350 emergency spells from a large teaching hospital in England between April 2004 and March 2014. Outcomes included 7-day, 30-day and in-hospital mortality rates. We conducted probit regressions to estimate the impact on the absolute difference in the risk of mortality of two key predictors: (1) admission on weekends (19:00 Friday to 06:59 Monday); and (2) night-time admission (19:00 to 06:59). Logistic regressions were used to estimate ORs for relative mortality risk differences.

**Results:**

Crude 30-day mortality rate decreased from 6.6% in 2004/2005 to 5.2% in 2013/2014. Adjusted mortality risk was elevated for all out-of-hours periods. The highest risk was associated with admission on weekend night-times: 30-day mortality increased by 0.6 percentage points (adjusted OR: 1.17, 95% CI 1.10 to 1.25), 7-day mortality by 0.5 percentage points (adjusted OR: 1.23, 95% CI 1.12 to 1.34) and in-hospital mortality by 0.5 percentage points (adjusted OR: 1.14, 95% CI 1.08 to 1.21) compared with admission on weekday daytimes. Weekend night-time admission was associated with increased mortality risk in 9 out of 10 years, but this was only statistically significant (p<0.05) in 5 out of 10 years.

**Conclusions:**

There is an increased risk of mortality for patients admitted as emergencies both on weekends and during the night-time. These effects are additive, so that the greatest risk of mortality occurs in patients admitted during the night on weekends. This increased risk appears to be consistent over time, but the effects are small and are not statistically significant in individual hospitals in every year.

## Introduction

Admission to hospital on weekends is consistently associated with poorer patient outcomes, for both emergency and non-emergency conditions.^1–5^ Payers and policy makers have responded to these findings with healthcare system reform on the assumption that reduced service provision underlies the higher risk of adverse outcomes on weekends, and that more consistent hospital care throughout the week will reduce or remove the disparity.^6 7^ In England, evidence on the ‘weekend effect’ has been used to justify controversial attempts to introduce a ‘seven day service’ across the National Health Service (NHS), an approach that is supported by evidence suggesting that emergency weekend admission is not associated with poorer outcomes when there is consistent access to early diagnosis and treatments.^8–10^

However, much of the evidence on the effect of weekend admission has been limited in key respects. First, it tends to be cross-sectional and there is therefore limited information on the trends in variations over time. Second, it is often based on routinely available data that provide the day but not the hour of admission. As a result, weekends have effectively been defined as 00:00 Saturday to 23:59 Sunday (thereby excluding Friday evenings and Monday mornings), and separate analysis of night-time admissions has not been possible. This is an important omission; if adverse patient outcomes are related to the different working practices and staff availability during weekends, then patients admitted during other out-of-hours periods are likely to be similarly affected. Some studies have used specialist data to address this issue, for example finding that at night there is an increased risk of mortality following in-hospital cardiac arrest^11^ or coronary artery bypass grafting^12^; an increased risk of in-hospital mortality if admitted to or discharged from intensive care units^13^; and a reduced chance of receiving timely intervention following stroke.^14 15^

For this study, we analysed emergency admissions to Salford Royal Foundation Trust (SRFT), a large teaching hospital in the Northwest of England providing a complete range of acute services, including specialist tertiary care. SRFT has maintained detailed electronic patient records since 2004, including precise time of admission, and has been a pioneer of extending normal hours of operation and providing enhanced weekend services. In 2011 it opened an ‘emergency village’, providing a consultant-led enhanced emergency service 7 days a week, and also extended services such as radiology, pathology and pharmacy across the weekend.

Using the enhanced data available from SRFT, we examined disparities in patient outcomes associated with out-of-hours admission across the full range of clinical specialties. We aimed to answer three key questions: (1) What is the size of the weekend effect when weekends are more appropriately defined (ie, 19:00 Friday to 06:59 Monday)? (2) Does the effect vary over time? (3) Is there a ‘night-time’ effect, with increased risk of all-cause mortality for patients admitted at night-time compared with daytime?

## Methods

### Data sources and variables

We extracted patient records from SRFT for 10 financial years, from April 2004 to March 2014. This data set included similar information to nationally available Hospital Statistics of Episode data: age, sex, time of admission, method and source of admission, primary and secondary diagnoses, specialty, procedures, time of discharge, destination on discharge, consultant codes, and the dates of in-hospital and out-of-hospital deaths. The original data were at consultant episode level (the period when a patient was under the treatment of a consultant), and we created hospital spells (the continuous stay in a single hospital from admission to discharge) by linking episode records from the time of admission to the time of discharge. We excluded maternity admissions throughout to facilitate temporal comparisons, as maternity services ceased to be provided in November 2011. We also excluded patients aged 16 years and under as SRFT does not provide paediatric services (maternal and paediatric cases attending A&E area were assessed and stabilised before being transferred to other treatment centres). For patients with multiple emergency admissions within the last 30 days of life, we excluded all but the first admission. The final sample included 246 350 emergency spells, defined as non-elective admissions.

Our primary outcome variable was mortality within 30 days of admission (either in or out of hospital). Subanalyses of 7-day and all in-hospital (within stay) mortality were conducted for robustness checks. Our main exposure variable was weekend admission. Our data set included the minute of admission, which allowed us to categorise admissions from 19:00 on 1 day to 06:59 the next day as ‘night’, and admissions from 19:00 on Friday to 06:59 on Monday as ‘weekend’, a more precise definition than previous studies, reflecting senior clinicians’ working patterns.^16^ We expected public holidays to have similar service arrangement as weekends, and we therefore identified emergency admissions on 56 public holidays in the 10-year period and grouped these with weekend admissions. To do this we first identified those holidays immediately before or after a weekend and linked the weekend and the holiday as a holiday period. Second, we included in all the public holidays the evening of the day before (from 19:00 to 23:59) and the morning of the day after (from 0:00 to 06:59). Patients admitted outside weekends or holidays were categorised as ‘weekday’ and analysed as a comparison group. Our secondary exposure was all admissions during out-of-hours periods including weekends and the nights of weekdays. We categorised patients by weekday daytime admission (07:00–18:59, Monday to Friday), weekday night-time admission (19:00–06:59, Monday to Thursday), weekend daytime admission (07:00–18:59, Saturday and Sunday) and weekend night-time admission (19:00–06:59, Friday to Sunday). Patients admitted during normal working hours (weekday-day group) were treated as the reference category.

We adjusted for patient case-mix by including variables available in the administrative hospital data. Collectively these variables provide a good fit for modelling the risk of mortality,^17^ although they do not include direct measures of severity of illness. We adjusted for patient demographics using age categories on admission, gender and ethnicity. Socioeconomic characteristics were measured using area deprivation for place of residence in quintiles, based on the 2010 Index of Multiple Deprivation at the lower layer super output area level.^18^ For each patient we calculated a Charlson Index score to account for the presence and the severity of comorbidities.^19^ We updated the weights of included conditions according to their association with the risk of mortality estimated using recent data from the UK.^20^ We made further adjustments for patient complexity using indicators for primary diagnosis summary groups,^21^ the total number of different diagnoses, the total number of different procedures during the admission, the use of palliative care during the spell and the total number of emergency admissions in the year prior to the index admission date. We also controlled for the method and source of admission, using the most common method or source as the reference category. We identified those patients discharged to other healthcare providers (both public and private) and adjusted this for 30-day and 7-day mortalities (this variable does not predict in-hospital death). We accounted for seasonal impact by including dummy variables for the month of admission. In the pooled analysis, we included year dummies to capture unobserved factors that varied by financial year.

### Statistical analysis

We fitted probit models to estimate the extent to which the probability of death was associated with our exposure variables, after controlling for observed patient characteristics. In the pooled analysis on the impact of out-of-hours admission, we assumed a latent propensity of death as a linear function of our exposure and controlling variables as outlined in equation (1).

$y_{i}^{*}=\;\alpha +\;\beta_{1}out-of-hours_{i}+\;\beta_{2}X_{i}+\;\gamma_{year\;}+\delta_{month}\;+\;\varepsilon_{i}$ (1),

where $y_{i}^{*}$ was the estimated propensity of death for spell *i*; $out-of-hours_{i}$ was a categorical variable indicating the type of out-of-hours period for admission *i*; $X_{i}$ was a vector of variables measuring patient demographics and complexity; $\gamma_{year}$ was a vector of dummies controlling for unobserved year-specific fixed effect; and $\delta_{month}$ was a vector of admission months adjusting for seasonal impact; the error term $\varepsilon_{i}$ was assumed to follow a standard normal distribution and was independent from the explanatory variables. The observed outcome for admission *i,* $Y_{i}$ depended on the value of $y_{i}^{*}$, so that

(2). $$Y_{i}=\;\left\{ \begin{array}{l} 1\;if\;y_{i\;}^{*}\&gt;0\;\\ 0\;if\;y_{i\;}^{*}\leq 0 \end{array}\right.$$

For the analysis of individual financial year, we removed the year dummies, $\gamma_{year}$, from equation (1), allowing the coefficients of explanatory variables to vary with time. We applied variance–covariance matrices clustered around consultant (first episode within each spell) to account for any correlation within management by the same consultant. In this non-linear model, the estimated coefficient of out-of-hours admission should not be interpreted as its effect size, because this also depends on the values of other predictors in the model. We therefore reported the average marginal effects (AME), which took into account the values of other predictors across the sample and computed the average changes in the risk of mortality associated with out-of-hours admission in absolute terms. We also estimated the same risk of mortality models using logistic regressions and reported ORs to show the relative differences in the mortality risk for comparability with previous studies. All analyses were conducted using Stata V.14.2.

## Results

There were a total of 246 350 emergency admissions for the study period from April 2004 to March 2014. Of the admissions, 5.9% resulted in death within 30 days, 2.6% in death within 7 days and 4.6% in death within the hospital stay. Crude 30-day mortality rates decreased over time, from 6.6% in 2004/2005 to 5.2% in 2013/2014 (online supplementary table and supplementary figure).

10.1136/bmjqs-2017-006784.supp1 Supplementary file 1

10.1136/bmjqs-2017-006784.supp2 Supplementary file 2

About a third of spells (81 621) were admitted during weekends and holidays. The demographic composition of weekend and weekday patients groups was similar (table 1). For both groups, average age was around 58.2 years, just under 50% of patients were male and 90% were white. The proportion of patients who lived in the most deprived fifth of areas was slightly higher for the weekend admission group (18.3% for weekend compared with 17.9% for weekday). Both groups were similar in terms of complexity: on average, each hospital stay had six different diagnoses, 1.5 procedures performed, one emergency admission in the year prior to the index admission date and a Charlson Index score of 5.

**Table 1:** Patient characteristics by admission time, 2004/2005–2013/2014

Admission time of the week	Weekday	Weekend*	Weekday-day	Weekday-night	Weekend-day*	Weekend-night*
	07:00 Monday– 18:59 Friday	19:00 Friday– 06:59 Monday	07:00–18:59 Monday–Friday	19:00–06:59 Monday–Thursday	07:00–18:59 Saturday and Sunday	19:00–06:59 Friday–Sunday
Admissions, n (%)	164 729 (66.87%)	81 621 (33.13%)	101 725 (41.29%)	63 004 (25.57%)	33 409 (13.56%)	48 212 (19.57%)
Age, mean (SD)	58.26 (21.17)	58.18 (21.82)	58.84 (20.93)	57.33 (21.53)	59.91 (21.69)	56.97 (21.84)
Gender, male (%)	79 736 (48.40%)	40 215 (49.27%)	48 742 (47.92%)	30 994 (49.19%)	16 264 (48.68%)	23 951 (49.68%)
Ethnicity, white (%)	148 215 (89.98%)	73 069 (89.52%)	91 784 (90.23%)	56 431 (89.57%)	29 964 (89.69%)	43 105 (89.41%)
Deprivation, most deprived quintile (%)	29 403 (17.85%)	14 973 (18.34%)	17 694 (17.39%)	11 709 (18.58%)	5884 (17.61%)	9089 (18.85%)
Diagnosis per spell, mean (SD)	5.96 (4.20)	6.10 (4.19)	5.85 (4.20)	6.13 (4.19)	6.07 (4.22)	6.12 (4.17)
Procedures per spell, mean (SD)	1.47 (2.50)	1.45 (2.54)	1.46 (2.46)	1.47 (2.56)	1.47 (2.51)	1.43 (2.55)
Previous emergency admission†, mean (SD)	0.95 (2.03)	1.01 (2.16)	0.89 (1.94)	1.04 (2.17)	0.97 (2.06)	1.03 (2.22)
Previous emergency admission‡, mean (SD)	2.46 (2.64)	2.60 (2.80)	2.36 (2.54)	2.60 (2.77)	2.53 (2.67)	2.64 (2.89)
Charlson Index, mean (SD)	5.03 (8.14)	5.10 (8.22)	5.01 (8.10)	5.08 (8.21)	5.31 (8.34)	4.96 (8.14)
Death in 30 days, n (%)	9366 (5.69%)	5180 (6.35%)	5765 (5.67%)	3601 (5.72%)	2241 (6.71%)	2939 (6.10%)
Death in 7 days, n (%)	3965 (2.41%)	2368 (2.90%)	2319 (2.28%)	1646 (2.61%)	1016 (3.04%)	1352 (2.80%)
Death in hospital, n (%)	7373 (4.48%)	4054 (4.97%)	4525 (4.45%)	2848 (4.52%)	1764 (5.28%)	2290 (4.75%)

*Including public holidays.

†In 365 days prior to the admission date.

‡For those having at least one emergency admission in the last 365 days.

Patients admitted at night were on average younger, and more likely to be male and from the most deprived fifth of areas compared with patients admitted during the day. Patients admitted at night also had on average more diagnosis codes and previous emergency admissions per spell than patients admitted during the day, although the absolute differences were marginal.

### Crude mortality for out-of-hours admissions

There were substantial differences in mortality rates between patients admitted on weekends compared with weekdays. The crude 30-day mortality rate for weekend admissions was 12.3% higher in relative terms than for weekday admissions (rates of 6.4% and 5.7%, respectively); the 7-day mortality rate was 20.8% higher (rates of 2.9% and 2.4%, respectively); and the in-hospital mortality rate was 11.1% higher (rates of 5.0% and 4.5%, respectively) (table 1).

Compared with normal working hours (daytime, weekdays), crude mortality rates were higher for patients admitted during all out-of-hours periods. Rates were highest for weekend daytimes. Compared with baseline crude 30-day mortality rates of 5.7%, mortality rates for patients admitted on weekend daytime were 17.5% higher (at 6.7%) and rates for weekend night-time were 7.0% higher (at 6.1%). We observed similar patterns for 7-day and in-hospital mortality.

### Adjusted mortality for out-of-hours admissions

Table 2 summarises the AMEs for the key predictors of mortality. Higher mortality was associated with increasing age, comorbidity, number of diagnoses and socioeconomic deprivation. Mortality was also higher for patients who were male, white (compared with black and Asian), received palliative care or were admitted via a general practitioner. Mortality was strongly associated with year of admission, with the risk declining over time.

**Table 2:** Adjusted risk of mortality 2004/2005–2013/2014, probit regressions with average marginal effects

	30-Day mortality		7-Day mortality		In-hospital mortality	
Variables	AME†	SE‡	AME	SE	AME	SE
Admission time						
Weekday	*Ref.*		*Ref.*		*Ref.*	
Weekend§	0.004***	(0.001)	0.003***	(0.001)	0.003***	(0.001)
Case-mix variables						
Age band 17–25	*Ref.*		*Ref.*		*Ref.*	
Age band 26–35	−0.001	(0.002)	−0.001	(0.001)	−0.003	(0.002)
Age band 36–45	0.005**	(0.002)	0.001	(0.001)	0.001	(0.002)
Age band 46–55	0.012***	(0.002)	0.005***	(0.001)	0.005**	(0.002)
Age band 56–65	0.025***	(0.003)	0.012***	(0.002)	0.015***	(0.003)
Age band 66–75	0.033***	(0.003)	0.015***	(0.002)	0.022***	(0.003)
Age band 76–85	0.051***	(0.003)	0.023***	(0.002)	0.038***	(0.003)
Age band 85+	0.079***	(0.004)	0.033***	(0.003)	0.067***	(0.005)
Gender, male	*Ref.*		*Ref.*		*Ref.*	
Gender, female	−0.003***	(0.001)	−0.001	(0.001)	−0.001	(0.001)
Gender, not stated	−0.005	(0.014)	−0.001	(0.011)	0.001	(0.016)
Ethnicity, white	*Ref.*		*Ref.*		*Ref.*	
Ethnicity, mixed	−0.013	(0.009)	−0.001	(0.006)	−0.010	(0.008)
Ethnicity, Asian	−0.015***	(0.004)	−0.007***	(0.002)	−0.013***	(0.003)
Ethnicity, black	−0.016***	(0.005)	−0.011***	(0.003)	−0.015***	(0.005)
Ethnicity, other	0.016***	(0.005)	0.013***	(0.004)	0.018***	(0.005)
Ethnicity, not stated	0.032***	(0.007)	0.022***	(0.005)	0.030***	(0.006)
IMD quintile-1 (most affluent)	*Ref.*		*Ref.*		*Ref.*	
IMD quintile-2	0.001	(0.002)	−0.000	(0.001)	−0.000	(0.001)
IMD quintile-3	0.002*	(0.001)	0.002	(0.001)	0.001	(0.001)
IMD quintile-4	0.002	(0.001)	−0.000	(0.001)	−0.000	(0.001)
IMD quintile-5 (most deprived)	0.004***	(0.001)	0.002*	(0.001)	0.003**	(0.001)
IMD quintile-missing	0.012***	(0.002)	0.005***	(0.001)	0.010***	(0.002)
Charlson Index	0.002***	(0.000)	0.001***	(0.000)	0.001***	(0.000)
Number of diagnosis	0.002***	(0.000)	−0.000	(0.000)	0.003***	(0.000)
Number of procedures	−0.001*	(0.000)	−0.002***	(0.000)	0.001***	(0.000)
Palliative care	0.150***	(0.013)	0.038***	(0.005)	0.107***	(0.009)
Number of emergency admissions in the previous 1 year (365 days)	−0.002***	(0.000)	−0.000**	(0.000)	−0.001***	(0.000)
Admission method, emergency A&E	*Ref.*		*Ref.*		*Ref.*	
Admission method, emergency transfer from other provider	−0.015***	(0.003)	−0.013***	(0.002)	−0.014***	(0.003)
Admission method, emergency domicile	−0.009	(0.017)	0.000	(0.000)	−0.026***	(0.009)
Admission method, emergency general practitioner refer	0.006**	(0.002)	−0.007***	(0.002)	0.002	(0.003)
Admission method, emergency outpatient	−0.018***	(0.003)	−0.018***	(0.002)	−0.017***	(0.003)
Admission method, emergency antenatal	0.025	(0.026)	0.010	(0.025)	0.000	(0.000)
Admission method, emergency postnatal	0.000	(0.000)	0.000	(0.000)	0.000	(0.000)
Admission method, non-emergency transfer from other provider	−0.015***	(0.004)	−0.015***	(0.003)	−0.016***	(0.004)
Transfer to other hospitals	−0.020***	(0.005)	−0.037***	(0.004)		
Financial year 2004/2005	*Ref.*		*Ref.*		*Ref.*	
Financial year 2005/2006	−0.012***	(0.004)	−0.002	(0.002)	−0.015***	(0.004)
Financial year 2006/2007	−0.011***	(0.003)	−0.003*	(0.002)	−0.020***	(0.003)
Financial year 2007/2008	−0.010**	(0.004)	0.001	(0.003)	−0.023***	(0.006)
Financial year 2008/2009	−0.023***	(0.004)	−0.005	(0.003)	−0.042***	(0.005)
Financial year 2009/2010	−0.029***	(0.005)	−0.006*	(0.003)	−0.048***	(0.005)
Financial year 2010/2011	−0.035***	(0.004)	−0.009**	(0.004)	−0.059***	(0.005)
Financial year 2011/2012	−0.039***	(0.004)	−0.010***	(0.004)	−0.064***	(0.005)
Financial year 2012/2013	−0.037***	(0.004)	−0.009***	(0.003)	−0.062***	(0.005)
Financial year 2013/2014	−0.042***	(0.004)	−0.011***	(0.004)	−0.067***	(0.005)
Diagnosis group dummies	*Yes*		*Yes*		*Yes*	
Admission source dummies	*Yes*		*Yes*		*Yes*	
Admission month dummies	*Yes*		*Yes*		*Yes*	
C statistic	0.88		0.87		0.90	
Pseudo-R^2^	0.28		0.23		0.31	
Observations	241 338		237 091		242 693	

†Average marginal effect. ***p<0.01, **p<0.05, *p<0.1.

‡Robust SEs corrected for clustering around consultant in parentheses.

§Including public holidays.

AME, average marginal effects; IMD, Index of Multiple Deprivation.

After adjusting for other factors, admission on weekends and public holidays had an independent and statistically significant association with 30-day mortality, with an estimated AME of 0.4 percentage points across the study period (adjusted OR: 1.10, 95% CI 1.06 to 1.15; online supplementary table). The risk of both 7-day and in-hospital mortality increased by 0.3 percentage points for admission on weekends and holidays (7-day adjusted OR: 1.12, 95% CI 1.07 to 1.18; in-hospital adjusted OR: 1.08, 95% CI 1.02 to 1.15), compared with weekday admission. Sensitivity analyses suggested that the effects of case-mix variables were robust across the three outcome measures.

In the pooled analysis, all out-of-hours admission periods were associated with higher risk of mortality after adjusting for confounders, compared with admission during normal working hours (table 3). Weekend night-time admission had the greatest impact on mortality: patients in this group had a 0.6 percentage points higher risk of death within 30 days (adjusted OR: 1.17, 95% CI 1.10 to 1.25; online supplementary table). Weekend daytime and weekday night-time admissions were also associated with increased risk of mortality within 30 days (weekend-day AME: 0.4 percentage points, adjusted OR: 1.11, 95% CI 1.04 to 1.18; weekday-night AME: 0.3 percentage points, adjusted OR: 1.09, 95% CI 1.01 to 1.17). Results for 7-day and in-hospital mortality were similar: patients admitted on weekend night-times had the highest risk of death, followed by weekend daytime and weekday night-time admissions.

**Table 3:** Adjusted risk of mortality 2004/2005–2013/2014, probit regressions with average marginal effects

	30-Day mortality		7-Day mortality		In-hospital mortality	
Variables	AME†	SE‡	AME	SE	AME	SE
Admission time						
Weekday day	*Ref.*		*Ref.*		*Ref.*	
Weekday night	0.003***	(0.001)	0.003***	(0.001)	0.003***	(0.001)
Weekend day§	0.004***	(0.001)	0.003***	(0.001)	0.003**	(0.002)
Weekend night§	0.006***	(0.001)	0.005***	(0.001)	0.005***	(0.001)
Case-mix variables						
Age band 17–25	*Ref.*		*Ref.*		*Ref.*	
Age band 26–35	−0.001	(0.002)	−0.001	(0.001)	−0.003	(0.002)
Age band 36–45	0.005**	(0.002)	0.001	(0.001)	0.001	(0.002)
Age band 46–55	0.012***	(0.002)	0.005***	(0.001)	0.005**	(0.002)
Age band 56–65	0.025***	(0.003)	0.012***	(0.002)	0.015***	(0.003)
Age band 66–75	0.033***	(0.003)	0.016***	(0.002)	0.022***	(0.003)
Age band 76–85	0.051***	(0.003)	0.023***	(0.002)	0.038***	(0.003)
Age band 85+	0.080***	(0.004)	0.033***	(0.003)	0.067***	(0.005)
Gender, male	*Ref.*		*Ref.*		*Ref.*	
Gender, female	−0.003***	(0.001)	−0.001	(0.001)	−0.001	(0.001)
Gender, not stated	−0.005	(0.014)	−0.001	(0.011)	0.001	(0.016)
Ethnicity, white	*Ref.*		*Ref.*		*Ref.*	
Ethnicity, mixed	−0.013	(0.009)	−0.001	(0.006)	−0.010	(0.008)
Ethnicity, Asian	−0.015***	(0.004)	−0.007***	(0.002)	−0.013***	(0.003)
Ethnicity, black	−0.016***	(0.005)	−0.011***	(0.003)	−0.015***	(0.005)
Ethnicity, other	0.016***	(0.005)	0.013***	(0.004)	0.018***	(0.005)
Ethnicity, not stated	0.032***	(0.007)	0.022***	(0.005)	0.030***	(0.006)
IMD quintile-1 (most affluent)	*Ref.*		*Ref.*		*Ref.*	
IMD quintile-2	0.001	(0.002)	−0.000	(0.001)	−0.000	(0.001)
IMD quintile-3	0.002*	(0.001)	0.002	(0.001)	0.001	(0.001)
IMD quintile-4	0.002	(0.001)	−0.000	(0.001)	−0.000	(0.001)
IMD quintile-5 (most deprived)	0.004***	(0.001)	0.002*	(0.001)	0.003**	(0.001)
IMD quintile-missing	0.012***	(0.002)	0.005***	(0.001)	0.010***	(0.002)
Charlson Index	0.002***	(0.000)	0.001***	(0.000)	0.001***	(0.000)
Number of diagnosis	0.002***	(0.000)	−0.000	(0.000)	0.003***	(0.000)
Number of procedures	−0.001*	(0.000)	−0.002***	(0.000)	0.001***	(0.000)
Palliative care	0.150***	(0.013)	0.038***	(0.005)	0.107***	(0.009)
Number of emergency admissions in the previous 1 year (365 days)	−0.002***	(0.000)	−0.000**	(0.000)	−0.001***	(0.000)
Admission method, emergency A&E	*Ref.*		*Ref.*		*Ref.*	
Admission method, emergency transfer from other provider	−0.015***	(0.003)	−0.013***	(0.002)	−0.013***	(0.003)
Admission method, emergency domicile	−0.008	(0.017)	0.000	(0.000)	−0.025***	(0.009)
Admission method, emergency general practitioner refer	0.007***	(0.002)	−0.006***	(0.002)	0.003	(0.003)
Admission method, emergency outpatient	−0.017***	(0.003)	−0.017***	(0.002)	−0.016***	(0.003)
Admission method, emergency antenatal	0.027	(0.026)	0.011	(0.025)	0.000	(0.000)
Admission method, emergency postnatal	0.000	(0.000)	0.000	(0.000)	0.000	(0.000)
Admission method, non-emergency transfer from other provider	−0.014***	(0.004)	−0.014***	(0.003)	−0.015***	(0.004)
Transfer to other hospitals	−0.020***	(0.005)	−0.037***	(0.004)		
Financial year 2004/2005	*Ref.*		*Ref.*		*Ref.*	
Financial year 2005/2006	−0.012***	(0.004)	−0.002	(0.002)	−0.015***	(0.004)
Financial year 2006/2007	−0.011***	(0.003)	−0.003*	(0.002)	−0.020***	(0.003)
Financial year 2007/2008	−0.010**	(0.004)	0.001	(0.003)	−0.023***	(0.006)
Financial year 2008/2009	−0.023***	(0.004)	−0.005	(0.003)	−0.042***	(0.005)
Financial year 2009/2010	−0.029***	(0.005)	−0.006*	(0.003)	−0.049***	(0.005)
Financial year 2010/2011	−0.036***	(0.004)	−0.009**	(0.004)	−0.059***	(0.005)
Financial year 2011/2012	−0.039***	(0.004)	−0.010***	(0.004)	−0.064***	(0.005)
Financial year 2012/2013	−0.038***	(0.004)	−0.009***	(0.003)	−0.063***	(0.005)
Financial year 2013/2014	−0.042***	(0.004)	−0.012***	(0.004)	−0.067***	(0.005)
Diagnosis group dummies	*Yes*		*Yes*		*Yes*	
Admission source dummies	*Yes*		*Yes*		*Yes*	
Admission month dummies	*Yes*		*Yes*		*Yes*	
C statistic	0.88		0.87		0.90	
Pseudo-R^2^	0.28		0.23		0.31	
Observations	241 338		237 091		242 693	

†Average marginal effect. ***p<0.01, **p<0.05, *p<0.1.

‡Robust SEs corrected for clustering around consultant in parentheses.

§Including public holidays.

AME, average marginal effects; IMD, Index of Multiple Deprivation.

### Variation in impact over time

The impact of weekend admission by financial year is reported in table 4. After case-mix adjustment, weekend admission was positively associated with 30-day mortality in 9 out of 10 years; however, this association was only statistically significant (p<0.05) in 3 out of the 10 years. The estimated AME was 0.6 percentage points for 2005/2006 (adjusted OR: 1.17, 95% CI 1.02 to 1.34; online supplementary table), 0.6 percentage points for 2008/2009 (adjusted OR: 1.14, 95% CI 1.02 to 1.27) and 0.9 percentage points for 2012/2013 (adjusted OR: 1.29, 95% CI 1.15 to 1.45). Seven-day mortality rates were elevated for weekend and holiday admissions in 2005/2006, 2011/2012 and 2012/2013 (p<0.05), whereas in-hospital mortality was not significantly associated with weekend admission in any year.

**Table 4:** Adjusted risk of mortality by financial year, probit regressions with average marginal effects

Year	N	Weekday	Weekend†	Weekday-day	Weekday-night	Weekend-day†	Weekend-night†
		07:00 Monday– 18:59 Friday	19:00 Friday– 06:59 Monday	07:00–18:59 Monday–Friday	19:00–06:59 Monday–Thursday	07:00–18:59 Saturday and Sunday	19:00–06:59 Friday–Sunday
30-Day mortality							
2004/2005	20 664	*Ref.*	0.004 (0.004)‡	*Ref.*	0.010*** (0.003)	0.004 (0.004)	0.011** (0.005)
2005/2006	20 254		0.006** (0.003)		0.002 (0.004)	0.008* (0.004)	0.005 (0.004)
2006/2007	20 991		0.003 (0.003)		0.010*** (0.004)	−0.000 (0.004)	0.012*** (0.004)
2007/2008	21 577		0.004 (0.002)		0.002 (0.003)	0.003 (0.004)	0.006* (0.003)
2008/2009	21 202		0.006** (0.002)		0.007** (0.003)	0.005 (0.004)	0.012*** (0.003)
2009/2010	22 115		−0.002 (0.003)		0.001 (0.003)	0.001 (0.006)	−0.003 (0.003)
2010/2011	23 642		0.006 (0.004)		−0.000 (0.004)	0.007 (0.005)	0.005 (0.006)
2011/2012	24 518		0.004* (0.002)		0.000 (0.003)	0.002 (0.004)	0.005** (0.002)
2012/2013	24 546		0.009*** (0.003)		0.001 (0.003)	0.010** (0.004)	0.009*** (0.003)
2013/2014	27 162		0.002 (0.002)		0.002 (0.002)	0.002 (0.003)	0.004 (0.003)
7-Day mortality							
2004/2005	18 526	*Ref.*	0.005* (0.003)	*Ref.*	0.009** (0.003)	0.007* (0.003)	0.009** (0.004)
2005/2006	17 646		0.005** (0.002)		0.002 (0.003)	0.009** (0.004)	0.003 (0.003)
2006/2007	18 826		0.001 (0.003)		0.009*** (0.003)	0.000 (0.004)	0.008** (0.003)
2007/2008	19 308		0.001 (0.002)		0.002 (0.003)	0.001 (0.004)	0.003 (0.003)
2008/2009	19 797		0.003 (0.002)		0.002 (0.002)	−0.000 (0.004)	0.008*** (0.003)
2009/2010	20 353		−0.000 (0.002)		0.006** (0.003)	0.003 (0.003)	0.002 (0.003)
2010/2011	21 906		0.002 (0.003)		0.004* (0.002)	0.002 (0.002)	0.004 (0.005)
2011/2012	20 610		0.007*** (0.002)		−0.002 (0.003)	0.005 (0.003)	0.007** (0.003)
2012/2013	22 127		0.005*** (0.002)		−0.001 (0.002)	0.005* (0.003)	0.005* (0.003)
2013/2014	24 716		0.001 (0.002)		0.004** (0.002)	0.002 (0.003)	0.003 (0.002)
In-hospital mortality							
2004/2005	20 454	*Ref.*	0.003 (0.003)	*Ref.*	0.009** (0.004)	0.003 (0.004)	0.008* (0.005)
2005/2006	20 231		0.004 (0.003)		0.004 (0.005)	0.004 (0.004)	0.007 (0.004)
2006/2007	20 605		0.002 (0.002)		0.008** (0.004)	0.000 (0.004)	0.010** (0.004)
2007/2008	21 763		0.003 (0.002)		−0.002 (0.003)	0.001 (0.004)	0.004 (0.003)
2008/2009	21 130		0.002 (0.003)		0.003 (0.003)	0.002 (0.004)	0.004 (0.003)
2009/2010	21 847		0.004 (0.003)		0.003 (0.003)	0.005 (0.006)	0.005* (0.003)
2010/2011	23 072		0.002 (0.002)		0.004 (0.003)	0.004 (0.003)	0.005 (0.004)
2011/2012	23 921		0.003* (0.002)		−0.002 (0.003)	0.002 (0.004)	0.003 (0.002)
2012/2013	23 808		0.004 (0.002)		−0.001 (0.002)	0.004 (0.003)	0.002 (0.003)
2013/2014	27 403		0.001 (0.002)		0.005** (0.002)	0.004 (0.003)	0.003 (0.003)

†Including public holidays.

‡Average marginal effect (***p<0.01, **p<0.05, *p<0.1), with robust SEs corrected for clustering around consultant in parentheses. Probit regressions adjusted for age, gender, ethnicity, deprivation, Charlson Index, number of diagnoses, number of procedures, palliative care, number of emergency admissions in last year, admission method, admission source, transfer-out dummy, admission month and primary diagnosis groups.

Results for out-of-hours admission by financial year are given in table 4. Admission during all three out-of-hours periods was associated with increased adjusted risk of mortality compared with weekday daytime admission in most years, although the pattern and size of impact varied and were not always statistically significant. Consistent with the pooled analysis, weekend night-time admission was associated with the greatest increased risk of mortality: there was a significantly increased risk of 30-day mortality in 2004/2005, 2006/2007, 2008/2009, 2011/2012 and 2012/2013. There was a significantly increased risk of mortality for weekend daytime admissions in 2012/2013 and for weekday night-time admissions in 2004/2005, 2006/2007 and 2008/2009.

## Discussion

In this study we analysed a quarter of a million emergency admissions to a large teaching hospital over a 10-year period. As with previous studies, we found that crude mortality rates were higher for patients admitted on weekends and holidays, compared with patients admitted on weekdays. We also found that risk-adjusted mortality rates were higher for patients admitted on weekends and for patients admitted at night-time. Compared with weekday daytime admissions, adjusted risk of 30-day mortality was 0.3 percentage points higher for weekday night-time admissions (adjusted OR: 1.09), 0.4 percentage points higher for weekend daytime admissions (adjusted OR: 1.11) and 0.6 percentage points higher for weekend night-time admissions (adjusted OR: 1.17).

### Strengths and weaknesses

This study addressed limitations in the existing literature in two main respects. First, by using exact time of admission, we could define weekends (and holidays) more precisely by including Friday evenings and Monday mornings, and could examine night-time admissions separately from daytime admissions for all clinical groups. The effect of night-time admission has been analysed mainly in disease-specific studies,^11–15^ with only few studies based on hospital-level data.^22^ Second, we were able to analyse the impact of our exposure variables longitudinally over a 10-year period and could quantify impacts in each financial year. We were therefore able to relax the assumption that risk factors have constant effects over time.

The study was subject to several limitations. First, it was based on patient records from a single hospital in the Northwest of England, although one that has been a pioneer in data collection and quality improvement. Heterogeneities between hospitals in terms of size, performance, financial status and patient demographics could affect the generalisability of our results. However, the focus on a single provider also confers advantages, including relatively consistent coding practices and population characteristics. The availability of longitudinal data for this provider enabled us to examine the effect of out-of-hours over time, but the restricted sample size limited the power of our study to detect differences in outcomes between groups, particularly for analyses of individual years. Second, we adjusted the risk of mortality for admission method and source but could not adjust for discharge destination due to the high correlation with the dependent variable (mortality). We therefore only accounted for the patients discharged to health providers (both public and private) and compared them with patients discharged to other destinations. Third, as with previous studies based on analysis of routinely collected hospital data, we had no direct measure of illness severity and our risk adjustment models were likely to be confounded by unmeasured severity. With the available information, we constructed several variables to adjust for patient complexity, including the Charlson Index score, the numbers of diagnoses and procedures in a spell. These variables might be under-recorded and less accurately coded during out-of-hours periods, which could create a potential bias.

### Main findings

Mortality rates for emergency admissions throughout the week declined over time, reflecting the wider fall in national mortality rates—attributable to net improvements in living conditions, lifestyles and healthcare—over the study period.^23^ In the context of these falling mortality rates over time, we found a persistently increased risk of mortality for patients admitted to hospital outside of normal hours—a pattern that has previously been found for stroke admissions.^24^ It might be expected that as overall mortality rates fall, rates for different time periods during the week would converge. We found no such temporal trend, but the smaller sample sizes for individual years mean that our study may have been underpowered to detect one. The reasons for both the improvements in survival for overall emergency admissions and the apparent persistence of elevated mortality rates for out-of-hours admissions warrant further investigation. As with the weekend effect itself, improving survival for admitted patients over time may reflect both changes in the admitted population and differences in quality of care, but the explanation must be consistent with the persistence of an increased risk of mortality for patients admitted out of hours. In the context of SRFT, the introduction of enhanced emergency services on weekends in 2011 had no apparent effect on excess mortality rates for out-of-hours admissions, although we did not formally test for such an effect. This has implications for current initiatives to introduce 7-day services across the NHS and the potential impact of these service changes on out-of-hours mortality.^25^

Our finding of increased risk of mortality at night-times across the full range of emergency admissions suggests that more patients are affected by service variations across the week than has been estimated in previous studies based on weekend/weekday comparisons.^1–5^ This finding is consistent with the results of studies on outcomes for cardiovascular disease,^11–15^ which suggest that patient outcomes are worse for acute events and invasive procedures occurring at night, not just on weekends. However, as with previous studies we cannot exclude the possibility that reduced capacity out-of-hours leads to the selection of a sicker patient population, and that this explains increased risk of mortality for both weekend and night-time admissions. Evidence from previous studies suggests there is a higher threshold for admission on weekends^26^ and that increased risk of mortality for out-of-hours admissions is reduced after adjusting for proxy^27^ measures of severity. If similar patterns apply at night-time, this would suggest that our findings reflect a reduced capacity for the hospital to admit less severely ill patients at nights and on weekends, rather than a reduced level of care at these times.

## Conclusion

In addition to patients admitted to hospital as an emergency on the weekend, patients admitted at night also experience higher mortality rates. These ‘weekend’ and ‘night-time’ effects are additive, with the highest risk of death for patients admitted at night on weekends. Recent UK policy has been moving towards the creation of a 7-day service,^28^ in part to address the perceived increased risk of adverse outcomes in patients admitted to hospital on weekends. This has generated conflict between a government attempting to increase weekend staffing levels while containing costs, and a medical profession resistant to these changes.^29^ For government policy to be consistent, it would also need to address adverse outcomes across all out-of-hours periods, including night-times on weekdays. Extending normal hours of operation could be beneficial if it leads to improved access and better patient outcomes, but clear evidence on cost-effectiveness will be needed to justify such major system changes.
