# Supplementary material for: Impact of out-of-hours admission on patient mortality: longitudinal analysis in a tertiary acute hospital
Source: BMJ Qual Saf. 2017 Sep 29;27(6):445–54. doi: 10.1136/bmjqs-2017-006784 (PMC5965349; doi:10.1136/bmjqs-2017-006784)
Supplement: Supplementary file 1 [file bmjqs-2017-006784supp001.docx]

**Appendix**

Table A1 Crude mortality rates, 2004/5 to 2013/14.

|  |  |  |  |  |  |  |  |  |
| --- | --- | --- | --- | --- | --- | --- | --- | --- |
| **Year** | **Admissions** | **Overall** | **Weekday** | **Weekend^1^** | **Weekday-day** | **Weekday-night** | **Weekend-day^1^** | **Weekend-night^1^** |
|  |  |  |  |  |  |  |  |  |
|  | **30-day mortality** | | | | | | | |
| 2004/05 | 22,001 | 6.61 | 6.45 | 6.96 | 6.32 | 6.72 | 7.28 | 6.69 |
| 2005/06 | 22,356 | 5.98 | 5.76 | 6.46 | 5.89 | 5.54 | 7.01 | 6.05 |
| 2006/07 | 22,946 | 6.40 | 6.19 | 6.85 | 5.95 | 6.62 | 6.73 | 6.93 |
| 2007/08 | 23,328 | 6.35 | 6.01 | 7.02 | 6.02 | 6.01 | 7.13 | 6.95 |
| 2008/09 | 23,194 | 6.36 | 6.04 | 7.05 | 5.90 | 6.25 | 7.27 | 6.89 |
| 2009/10 | 24,381 | 5.61 | 5.55 | 5.73 | 5.66 | 5.38 | 6.04 | 5.52 |
| 2010/11 | 25,821 | 5.91 | 5.72 | 6.27 | 5.59 | 5.90 | 6.96 | 5.80 |
| 2011/12 | 26,291 | 5.38 | 5.14 | 5.86 | 5.13 | 5.14 | 6.17 | 5.65 |
| 2012/13 | 26,276 | 5.64 | 5.34 | 6.20 | 5.29 | 5.42 | 6.74 | 5.84 |
| 2013/14 | 29,756 | 5.17 | 4.97 | 5.56 | 5.08 | 4.81 | 5.99 | 5.28 |
| Total | 246,350 | 5.90 | 5.69 | 6.35 | 5.67 | 5.72 | 6.71 | 6.10 |
|  |  |  |  |  |  |  |  |  |
|  | **7-day mortality** | | | | | | | |
| 2004/05 | 22,001 | 2.75 | 2.54 | 3.17 | 2.33 | 2.98 | 3.24 | 3.11 |
| 2005/06 | 22,356 | 2.74 | 2.56 | 3.14 | 2.56 | 2.56 | 3.55 | 2.83 |
| 2006/07 | 22,946 | 2.73 | 2.65 | 2.91 | 2.34 | 3.19 | 2.83 | 2.96 |
| 2007/08 | 23,328 | 2.88 | 2.72 | 3.20 | 2.61 | 2.89 | 3.29 | 3.13 |
| 2008/09 | 23,194 | 2.75 | 2.54 | 3.19 | 2.45 | 2.68 | 3.04 | 3.30 |
| 2009/10 | 24,381 | 2.44 | 2.33 | 2.66 | 2.14 | 2.63 | 2.86 | 2.53 |
| 2010/11 | 25,821 | 2.57 | 2.50 | 2.72 | 2.32 | 2.75 | 2.84 | 2.64 |
| 2011/12 | 26,291 | 2.37 | 2.11 | 2.87 | 2.10 | 2.13 | 3.20 | 2.66 |
| 2012/13 | 26,276 | 2.46 | 2.24 | 2.88 | 2.14 | 2.39 | 3.10 | 2.74 |
| 2013/14 | 29,756 | 2.19 | 2.04 | 2.49 | 1.91 | 2.22 | 2.58 | 2.43 |
| Total | 246,350 | 2.57 | 2.41 | 2.90 | 2.28 | 2.61 | 3.04 | 2.80 |
|  |  |  |  |  |  |  |  |  |
|  | **In-hospital mortality** | | | | | | | |
| 2004/05 | 22,001 | 5.93 | 5.81 | 6.19 | 5.83 | 5.77 | 6.57 | 5.87 |
| 2005/06 | 22,356 | 5.35 | 5.19 | 5.69 | 5.17 | 5.23 | 5.95 | 5.49 |
| 2006/07 | 22,946 | 5.30 | 5.17 | 5.58 | 4.91 | 5.61 | 5.49 | 5.64 |
| 2007/08 | 23,328 | 5.22 | 4.98 | 5.71 | 5.00 | 4.93 | 6.05 | 5.48 |
| 2008/09 | 23,194 | 5.07 | 4.85 | 5.51 | 4.73 | 5.05 | 5.86 | 5.26 |
| 2009/10 | 24,381 | 4.52 | 4.29 | 4.99 | 4.28 | 4.31 | 5.20 | 4.86 |
| 2010/11 | 25,821 | 4.34 | 4.22 | 4.57 | 4.05 | 4.45 | 4.90 | 4.36 |
| 2011/12 | 26,291 | 3.69 | 3.51 | 4.05 | 3.53 | 3.48 | 4.41 | 3.82 |
| 2012/13 | 26,276 | 3.92 | 3.79 | 4.16 | 3.78 | 3.81 | 4.45 | 3.97 |
| 2013/14 | 29,756 | 3.68 | 3.50 | 4.03 | 3.47 | 3.55 | 4.45 | 3.75 |
| Total | 246,350 | 4.64 | 4.48 | 4.97 | 4.45 | 4.52 | 5.28 | 4.75 |
| 1. Including public holidays. |  |  |  |  |  |  |  |  |

Figure A1 Crude 30-day mortality rates, 2004/05 to 2013/14.

Table A2 Adjusted risk of mortality 2004/05-2013/14, logistic regressions with odds ratios.

|  | **30-day mortality** | | **7-day mortality** | | **In-hospital mortality** | |
| --- | --- | --- | --- | --- | --- | --- |
| **Variables** | Odds ratio^2^ | 95% CI^3^ | Odds ratio | 95% CI | Odds ratio | 95% CI |
|  |  |  |  |  |  |  |
| ***Admission time*** |  |  |  |  |  |  |
| Weekday | *Ref.* |  | *Ref.* |  | *Ref.* |  |
| Weekend^1^ | 1.104 | [1.057, 1.154] | 1.122 | [1.069, 1.179] | 1.083 | [1.021, 1.149] |
|  |  |  |  |  |  |  |
| ***Case-mix variables*** |  |  |  |  |  |  |
| Age band 17-25 | Ref. |  | Ref. |  | Ref. |  |
| Age band 26-35 | 0.962 | [0.685, 1.349] | 0.890 | [0.639, 1.241] | 0.842 | [0.628, 1.128] |
| Age band 36-45 | 1.484 | [1.086, 2.028] | 1.247 | [0.931, 1.671] | 1.216 | [0.913, 1.619] |
| Age band 46-55 | 2.161 | [1.522, 3.069] | 1.719 | [1.218, 2.425] | 1.585 | [1.144, 2.196] |
| Age band 56-65 | 3.362 | [2.240, 5.048] | 2.608 | [1.666, 4.082] | 2.357 | [1.582, 3.511] |
| Age band 66-75 | 4.139 | [2.773, 6.177] | 3.081 | [1.998, 4.752] | 3.025 | [2.062, 4.436] |
| Age band 76-85 | 6.074 | [4.076, 9.050] | 4.100 | [2.663, 6.312] | 4.518 | [3.073, 6.641] |
| Age band 85+ | 9.454 | [6.312, 14.159] | 5.429 | [3.416, 8.629] | 7.753 | [5.137, 11.699] |
|  |  |  |  |  |  |  |
| Gender-male | *Ref.* |  | *Ref.* |  | *Ref.* |  |
| Gender-female | 0.934 | [0.880, 0.992] | 0.980 | [0.920, 1.044] | 0.990 | [0.942, 1.040] |
| Gender-not stated | 0.858 | [0.401, 1.832] | 0.962 | [0.377, 2.458] | 1.037 | [0.501, 2.147] |
|  |  |  |  |  |  |  |
| Ethnicity-White | *Ref.* |  | *Ref.* |  | *Ref.* |  |
| Ethnicity-mixed | 0.677 | [0.341, 1.344] | 0.956 | [0.513, 1.784] | 0.708 | [0.364, 1.376] |
| Ethnicity-Asian | 0.614 | [0.462, 0.816] | 0.655 | [0.464, 0.926] | 0.634 | [0.481, 0.836] |
| Ethnicity-Black | 0.555 | [0.376, 0.818] | 0.469 | [0.260, 0.848] | 0.532 | [0.339, 0.835] |
| Ethnicity-other | 1.448 | [1.148 , 1.825] | 1.646 | [1.262, 2.146] | 1.534 | [1.234, 1.906] |
| Ethnicity-not stated | 1.930 | [1.512, 2.463] | 2.122 | [1.636, 2.751] | 2.035 | [1.613, 2.567] |
|  |  |  |  |  |  |  |
| IMD quintile-1 (most affluent) | *Ref.* |  | *Ref.* |  | *Ref.* |  |
| IMD quintile-2 | 1.010 | [0.930, 1.096] | 0.984 | [0.909, 1.066] | 0.972 | [0.900, 1.051] |
| IMD quintile-3 | 1.045 | [0.980, 1.115] | 1.059 | [0.970, 1.156] | 1.007 | [0.947, 1.071] |
| IMD quintile-4 | 1.033 | [0.963, 1.108] | 0.971 | [0.886, 1.063] | 0.972 | [0.906, 1.043] |
| IMD quintile-5 (most deprived) | 1.090 | [1.025, 1.159] | 1.066 | [0.980, 1.159] | 1.064 | [0.998, 1.135] |
| IMD quintile-missing | 1.300 | [1.194, 1.417] | 1.219 | [1.094, 1.359] | 1.291 | [1.189, 1.401] |
|  |  |  |  |  |  |  |
| Charlson index | 1.040 | [1.036, 1.045] | 1.037 | [1.032, 1.042] | 1.034 | [1.030, 1.039] |
|  |  |  |  |  |  |  |
| No. of diagnosis | 1.037 | [1.021, 1.053] | 0.987 | [0.967, 1.008] | 1.094 | [1.076, 1.114] |
| No. of procedures | 0.977 | [0.960, 0.994] | 0.911 | [0.876, 0.948] | 1.035 | [1.016, 1.054] |
| Palliative care | 6.744 | [5.558, 8.182] | 2.831 | [2.268, 3.534] | 5.608 | [4.610, 6.823] |
| No. of emergency admissions in the previous 1 year (365 days) | 0.953 | [0.936, 0.969] | 0.976 | [0.958, 0.995] | 0.967 | [0.948, 0.987] |
|  |  |  |  |  |  |  |
| Admission method-emergency A+E | *Ref.* |  | *Ref.* |  | *Ref.* |  |
| Admission method-emergency transfer from other provider | 0.642 | [0.544, 0.756] | 0.479 | [0.378, 0.606] | 0.655 | [0.543, 0.791] |
| Admission method-emergency domicile | 0.824 | [0.339, 2.005] | 1.000 | [1.000, 1.000] | 0.454 | [0.177, 1.165] |
| Admission method-emergency GP refer | 1.124 | [1.010, 1.252] | 0.702 | [0.581, 0.848] | 1.038 | [0.912, 1.182] |
| Admission method-emergency outpatient | 0.591 | [0.494, 0.708] | 0.321 | [0.225, 0.457] | 0.575 | [0.452, 0.732] |
| Admission method-emergency ante natal | 1.764 | [0.583, 5.331] | 1.579 | [0.243, 10.251] | 1.000 | [1.000, 1.000] |
| Admission method-emergency post natal | 1.000 | [1.000, 1.000] | 1.000 | [1.000, 1.000] | 1.000 | [1.000, 1.000] |
| Admission method-non-emergency transfer from other provider | 0.661 | [0.518, 0.843] | 0.441 | [0.323, 0.603] | 0.623 | [0.487, 0.796] |
|  |  |  |  |  |  |  |
| Transfer to other hospitals | 0.584 | [0.471, 0.726] | 0.177 | [0.137, 0.229] |  |  |
|  |  |  |  |  |  |  |
| Financial year 2004/05 | *Ref.* |  | *Ref.* |  | *Ref.* |  |
| Financial year 2005/06 | 0.788 | [0.665, 0.933] | 0.914 | [0.779, 1.072] | 0.754 | [0.656, 0.866] |
| Financial year 2006/07 | 0.805 | [0.707, 0.916] | 0.913 | [0.804, 1.037] | 0.681 | [0.603, 0.767] |
| Financial year 2007/08 | 0.812 | [0.690, 0.956] | 1.072 | [0.847, 1.357] | 0.626 | [0.507, 0.771] |
| Financial year 2008/09 | 0.615 | [0.521, 0.726] | 0.855 | [0.679, 1.078] | 0.398 | [0.321, 0.493] |
| Financial year 2009/10 | 0.533 | [0.440, 0.646] | 0.829 | [0.643, 1.070] | 0.335 | [0.266, 0.423] |
| Financial year 2010/11 | 0.453 | [0.369, 0.555] | 0.744 | [0.533, 1.038] | 0.233 | [0.184, 0.295] |
| Financial year 2011/12 | 0.403 | [0.326, 0.498] | 0.698 | [0.510, 0.955] | 0.192 | [0.154, 0.240] |
| Financial year 2012/13 | 0.420 | [0.346, 0.511] | 0.720 | [0.548, 0.945] | 0.202 | [0.162, 0.252] |
| Financial year 2013/14 | 0.367 | [0.293, 0.460] | 0.655 | [0.478, 0.897] | 0.169 | [0.130, 0.220] |
|  |  |  |  |  |  |  |
| Diagnosis group dummies | *Yes* |  | *Yes* |  | *Yes* |  |
| Admission source dummies | *Yes* |  | *Yes* |  | *Yes* |  |
| Admission month dummies | *Yes* |  | *Yes* |  | *Yes* |  |
|  |  |  |  |  |  |  |
| **C statistic** | 0.88 |  | 0.88 |  | 0.90 |  |
| **Pseudo R2** | 0.28 |  | 0.23 |  | 0.31 |  |
| **Observations** | 241,338 |  | 237,091 |  | 242,693 |  |
|  |  |  |  |  |  |  |

1. Including public holidays.

2. Estimated by logistic regression.

3. 95% confidence interval corrected for clustering around consultant.

Table A3 Adjusted risk of mortality 2004/05-2013/14, logistic regressions with odds ratios.

|  | **30-day mortality** | | **7-day mortality** | | **In-hospital mortality** | |
| --- | --- | --- | --- | --- | --- | --- |
| **Variables** | Odds ratio^2^ | 95% CI^3^ | Odds ratio | 95% CI | Odds ratio | 95% CI |
|  |  |  |  |  |  |  |
| ***Admission time*** |  |  |  |  |  |  |
| Weekday day | *Ref.* |  | *Ref.* |  | *Ref.* |  |
| Weekday night | 1.087 | [1.012, 1.167] | 1.160 | [1.061, 1.268] | 1.088 | [1.029, 1.150] |
| Weekend day^1^ | 1.110 | [1.041, 1.183] | 1.155 | [1.069, 1.249] | 1.095 | [1.010, 1.187] |
| Weekend night^1^ | 1.168 | [1.095, 1.245] | 1.225 | [1.121, 1.338] | 1.140 | [1.077, 1.208] |
|  |  |  |  |  |  |  |
| ***Case-mix variables*** |  |  |  |  |  |  |
| Age band 17-25 | Ref. |  | Ref. |  | Ref. |  |
| Age band 26-35 | 0.963 | [0.687, 1.350] | 0.891 | [0.640, 1.242] | 0.843 | [0.629, 1.129] |
| Age band 36-45 | 1.485 | [1.087, 2.029] | 1.247 | [0.931, 1.672] | 1.216 | [0.913, 1.619] |
| Age band 46-55 | 2.163 | [1.523, 3.073] | 1.720 | [1.219, 2.428] | 1.585 | [1.144, 2.197] |
| Age band 56-65 | 3.373 | [2.247, 5.061] | 2.620 | [1.675, 4.098] | 2.363 | [1.586, 3.521] |
| Age band 66-75 | 4.155 | [2.786, 6.195] | 3.100 | [2.012, 4.777] | 3.035 | [2.069, 4.451] |
| Age band 76-85 | 6.095 | [4.094, 9.073] | 4.122 | [2.680, 6.338] | 4.532 | [3.084, 6.660] |
| Age band 85+ | 9.502 | [6.354, 14.208] | 5.473 | [3.453, 8.673] | 7.789 | [5.165, 11.747] |
|  |  |  |  |  |  |  |
| Gender-male | *Ref.* |  | *Ref.* |  | *Ref.* |  |
| Gender-female | 0.934 | [0.880, 0.991] | 0.979 | [0.919, 1.043] | 0.990 | [0.942, 1.040] |
| Gender-not stated | 0.858 | [0.401, 1.834] | 0.962 | [0.372, 2.487] | 1.036 | [0.500, 2.148] |
|  |  |  |  |  |  |  |
| Ethnicity-White | *Ref.* |  | *Ref.* |  | *Ref.* |  |
| Ethnicity-mixed | 0.677 | [0.339, 1.350] | 0.955 | [0.509, 1.790] | 0.708 | [0.363, 1.379] |
| Ethnicity-Asian | 0.613 | [0.462, 0.814] | 0.654 | [0.463, 0.925] | 0.633 | [0.480, 0.835] |
| Ethnicity-Black | 0.555 | [0.376, 0.819] | 0.470 | [0.260, 0.850] | 0.532 | [0.338, 0.838] |
| Ethnicity-other | 1.443 | [1.147, 1.815] | 1.636 | [1.259, 2.126] | 1.528 | [1.230, 1.900] |
| Ethnicity-not stated | 1.932 | [1.513, 2.468] | 2.125 | [1.637, 2.758] | 2.037 | [1.614, 2.572] |
|  |  |  |  |  |  |  |
| IMD quintile-1 (most affluent) | *Ref.* |  | *Ref.* |  | *Ref.* |  |
| IMD quintile-2 | 1.009 | [0.930, 1.095] | 0.984 | [0.910, 1.066] | 0.972 | [0.900, 1.051] |
| IMD quintile-3 | 1.045 | [0.980, 1.115] | 1.059 | [0.970, 1.156] | 1.007 | [0.947, 1.070] |
| IMD quintile-4 | 1.032 | [0.962, 1.107] | 0.970 | [0.886, 1.062] | 0.972 | [0.906, 1.043] |
| IMD quintile-5 (most deprived) | 1.089 | [1.024, 1.159] | 1.065 | [0.979, 1.158] | 1.063 | [0.997, 1.134] |
| IMD quintile-missing | 1.298 | [1.191, 1.414] | 1.216 | [1.091, 1.356] | 1.289 | [1.187, 1.399] |
|  |  |  |  |  |  |  |
| Charlson index | 1.040 | [1.036, 1.045] | 1.037 | [1.032, 1.042] | 1.034 | [1.030, 1.039] |
|  |  |  |  |  |  |  |
| No. of diagnosis | 1.037 | [1.021, 1.053] | 0.987 | [0.967, 1.008] | 1.094 | [1.076, 1.114] |
| No. of procedures | 0.976 | [0.960, 0.993] | 0.911 | [0.876, 0.948] | 1.034 | [1.016, 1.053] |
| Palliative care | 6.745 | [5.562, 8.180] | 2.829 | [2.267, 3.531] | 5.608 | [4.610, 6.822] |
| No. of emergency admissions in the previous 1 year (365 days) | 0.953 | [0.936, 0.969] | 0.976 | [0.958, 0.995] | 0.967 | [0.948, 0.987] |
|  |  |  |  |  |  |  |
| Admission method-emergency A+E | *Ref.* |  | *Ref.* |  | *Ref.* |  |
| Admission method-emergency transfer from other provider | 0.653 | [0.554, 0.770] | 0.493 | [0.389, 0.624] | 0.666 | [0.551, 0.805] |
| Admission method-emergency domicile | 0.832 | [0.341, 2.033] | 1.000 | [1.000, 1.000] | 0.459 | [0.179, 1.177] |
| Admission method-emergency GP refer | 1.145 | [1.022, 1.282] | 0.725 | [0.597, 0.881] | 1.057 | [0.927, 1.206] |
| Admission method-emergency outpatient | 0.605 | [0.506, 0.724] | 0.335 | [0.236, 0.476] | 0.589 | [0.464, 0.749] |
| Admission method-emergency ante natal | 1.814 | [0.611, 5.388] | 1.636 | [0.260, 10.278] | 1.000 | [1.000, 1.000] |
| Admission method-emergency post natal | 1.000 | [1.000, 1.000] | 1.000 | [1.000, 1.000] | 1.000 | [1.000, 1.000] |
| Admission method-non-emergency transfer from other provider | 0.673 | [0.526, 0.860] | 0.455 | [0.333, 0.621] | 0.633 | [0.495, 0.810] |
|  |  |  |  |  |  |  |
| Transfer to other hospitals | 0.583 | [0.470, 0.725] | 0.176 | [0.137, 0.228] |  |  |
|  |  |  |  |  |  |  |
| Financial year 2004/05 | *Ref.* |  | *Ref.* |  | *Ref.* |  |
| Financial year 2005/06 | 0.786 | [0.663, 0.931] | 0.910 | [0.776, 1.069] | 0.752 | [0.654, 0.864] |
| Financial year 2006/07 | 0.802 | [0.704, 0.914] | 0.908 | [0.801, 1.031] | 0.678 | [0.601, 0.765] |
| Financial year 2007/08 | 0.809 | [0.687, 0.952] | 1.066 | [0.842, 1.350] | 0.623 | [0.505, 0.769] |
| Financial year 2008/09 | 0.613 | [0.519, 0.724] | 0.852 | [0.675, 1.074] | 0.396 | [0.320, 0.491] |
| Financial year 2009/10 | 0.531 | [0.439, 0.643] | 0.825 | [0.639, 1.064] | 0.334 | [0.265, 0.421] |
| Financial year 2010/11 | 0.450 | [0.368, 0.552] | 0.738 | [0.530, 1.026] | 0.232 | [0.183, 0.293] |
| Financial year 2011/12 | 0.402 | [0.326, 0.495] | 0.693 | [0.508, 0.946] | 0.192 | [0.154, 0.239] |
| Financial year 2012/13 | 0.418 | [0.345, 0.507] | 0.715 | [0.545, 0.938] | 0.201 | [0.162, 0.251] |
| Financial year 2013/14 | 0.366 | [0.293, 0.458] | 0.651 | [0.476, 0.891] | 0.169 | [0.130, 0.219] |
|  |  |  |  |  |  |  |
| Diagnosis group dummies | *Yes* |  | *Yes* |  | *Yes* |  |
| Admission source dummies | *Yes* |  | *Yes* |  | *Yes* |  |
| Admission month dummies | *Yes* |  | *Yes* |  | *Yes* |  |
|  |  |  |  |  |  |  |
| **C statistic** | 0.88 |  | 0.88 |  | 0.90 |  |
| **Pseudo R2** | 0.28 |  | 0.24 |  | 0.31 |  |
| **Observations** | 241,338 |  | 237,091 |  | 242,693 |  |
|  |  |  |  |  |  |  |
| 1. Including public holidays.  2. Estimated by logistic regression.  3. 95% confidence interval corrected for clustering around consultant. |  |  |  |  |  |  |

Table A4 Adjusted risk of mortality by financial year, logistic regressions with odds ratios.

| **Year** | **N** | **Weekday** | **Weekend^1^** | **Weekday-day** | **Weekday-night** | **Weekend-day^1^** | **Weekend-night^1^** |
| --- | --- | --- | --- | --- | --- | --- | --- |
|  |  | 7am Mon -  6.59pm Fri | 7pm Fri -  6.59am Mon | 7am - 6.59pm  Mon - Fri | 7pm - 6.59am  Mon - Thu | 7am - 6.59pm  Sat & Sun | 7pm – 6.59am  Fri - Sun |
|  |  | | | | | | |
|  | **30-day mortality** | | | | | | |
| 2004/05 | 20,664 | *Ref.* | 1.068 [0.917, 1.244]^2^ | *Ref.* | 1.250 [1.080, 1.446] | 1.078 [0.891, 1.303] | 1.223 [0.984, 1.521] |
| 2005/06 | 20,254 |  | 1.166 [1.015, 1.340] |  | 1.064 [0.859, 1.318] | 1.237 [1.021, 1.499] | 1.157 [0.943, 1.420] |
| 2006/07 | 20,991 |  | 1.064 [0.941, 1.204] |  | 1.224 [1.048, 1.430] | 0.997 [0.812, 1.225] | 1.285 [1.092, 1.511] |
| 2007/08 | 21,577 |  | 1.099 [0.996, 1.212] |  | 1.049 [0.913, 1.207] | 1.079 [0.923, 1.260] | 1.152 [1.002, 1.324] |
| 2008/09 | 21,202 |  | 1.137 [1.022, 1.265] |  | 1.170 [1.012, 1.351] | 1.093 [0.923, 1.295] | 1.314 [1.134, 1.522] |
| 2009/10 | 22,115 |  | 0.970 [0.829, 1.134] |  | 1.030 [0.870, 1.220] | 1.026 [0.743, 1.417] | 0.951 [0.817, 1.107] |
| 2010/11 | 23,642 |  | 1.145 [0.947, 1.384] |  | 0.990 [0.817, 1.200] | 1.188 [0.945, 1.492] | 1.105 [0.821, 1.487] |
| 2011/12 | 24,518 |  | 1.078 [0.970, 1.199] |  | 1.011 [0.872, 1.172] | 1.037 [0.836, 1.287] | 1.121 [0.984, 1.277] |
| 2012/13 | 24,546 |  | 1.287 [1.146, 1.446] |  | 1.026 [0.888, 1.185] | 1.330 [1.114, 1.587] | 1.282 [1.088, 1.510] |
| 2013/14 | 27,162 |  | 1.040 [0.934, 1.158] |  | 1.052 [0.947, 1.168] | 1.033 [0.870, 1.227] | 1.084 [0.936, 1.255] |
|  |  |  |  |  |  |  |  |
|  | **7-day mortality** | | | | | | |
| 2004/05 | 18,526 | *Ref.* | 1.195 [0.986, 1.448] | *Ref.* | 1.350 [1.071, 1.702] | 1.304 [1.009, 1.685] | 1.361 [1.026, 1.806] |
| 2005/06 | 17,646 |  | 1.181 [1.008, 1.385] |  | 1.079 [0.867, 1.344] | 1.314 [1.017, 1.698] | 1.137 [0.920, 1.406] |
| 2006/07 | 18,826 |  | 1.038 [0.856, 1.259] |  | 1.357 [1.115, 1.651] | 1.020 [0.750, 1.386] | 1.310 [1.077, 1.593] |
| 2007/08 | 19,308 |  | 1.041 [0.899, 1.205] |  | 1.085 [0.882, 1.335] | 1.055 [0.818, 1.362] | 1.092 [0.904, 1.318] |
| 2008/09 | 19,797 |  | 1.137 [0.968, 1.336] |  | 1.083 [0.890, 1.317] | 1.003 [0.760, 1.323] | 1.321 [1.067, 1.634] |
| 2009/10 | 20,353 |  | 1.003 [0.845, 1.191] |  | 1.304 [1.057, 1.609] | 1.149 [0.870, 1.518] | 1.109 [0.875, 1.404] |
| 2010/11 | 21,906 |  | 1.069 [0.850, 1.344] |  | 1.182 [0.973, 1.438] | 1.083 [0.870, 1.349] | 1.209 [0.805, 1.816] |
| 2011/12 | 20,610 |  | 1.289 [1.093, 1.520] |  | 0.944 [0.740, 1.205] | 1.234 [0.964, 1.581] | 1.275 [0.959, 1.695] |
| 2012/13 | 22,127 |  | 1.271 [1.100, 1.468] |  | 1.004 [0.838, 1.203] | 1.289 [1.018, 1.633] | 1.262 [1.035, 1.539] |
| 2013/14 | 24,716 |  | 1.021 [0.855, 1.220] |  | 1.230 [1.011, 1.496] | 1.093 [0.815, 1.464] | 1.135 [0.901, 1.430] |
|  |  |  |  |  |  |  |  |
|  | **In-hospital mortality** | | | | | | |
| 2004/05 | 20,454 | *Ref.* | 1.059 [0.922, 1.216] | *Ref.* | 1.211 [1.031, 1.423] | 1.099 [0.923, 1.309] | 1.160 [0.945, 1.425] |
| 2005/06 | 20,231 |  | 1.122 [0.969, 1.300] |  | 1.107 [0.880, 1.394] | 1.119 [0.942, 1.330] | 1.024 [0.977, 1.485] |
| 2006/07 | 20,605 |  | 1.055 [0.939, 1.185] |  | 1.207 [0.984, 1.480] | 0.990 [0.809, 1.213] | 1.261 [1.045, 1.521] |
| 2007/08 | 21,763 |  | 1.081 [0.960, 1.217] |  | 0.969 [0.841, 1.116] | 1.038 [0.865, 1.246] | 1.091 [0.929, 1.280] |
| 2008/09 | 21,130 |  | 1.038 [0.897, 1.201] |  | 1.069 [0.932, 1.226] | 1.029 [0.850, 1.244] | 1.098 [0.926, 1.301] |
| 2009/10 | 21,847 |  | 1.127 [0.960, 1.323] |  | 1.088 [0.942, 1.256] | 1.153 [0.836, 1.590] | 1.178 [1.002, 1.383] |
| 2010/11 | 23,072 |  | 1.075 [0.939, 1.231] |  | 1.120 [0.911, 1.377] | 1.112 [0.948, 1.306] | 1.146 [0.900, 1.459] |
| 2011/12 | 23,921 |  | 1.090 [0.966, 1.230] |  | 0.931 [0.777, 1.115] | 1.057 [0.834, 1.340] | 1.056 [0.915, 1.219] |
| 2012/13 | 23,808 |  | 1.131 [0.990, 1.291] |  | 0.958 [0.839, 1.094] | 1.145 [0.939, 1.398] | 1.085 [0.906, 1.299] |
| 2013/14 | 27.403 |  | 1.032 [0.902, 1.180] |  | 1.187 [1.014, 1.390] | 1.175 [0.944, 1.462] | 1.064 [0.869, 1.302] |

1. Including public holidays.

2. Odds ratio estimated by logistic regression with 95% confidence interval corrected for clustering around consultant in brackets. Logistic regressions adjusted

for age, gender, ethnicity, deprivation, Charlson index, number of diagnoses, number of procedures, palliative care, number of emergency admissions in last

year, admission method, admission source, transfer-out dummy, admission month and primary diagnosis groups.
