# Supplementary figures and images for: Impact of out-of-hours admission on patient mortality: longitudinal analysis in a tertiary acute hospital
Source: BMJ Qual Saf. 2017 Sep 29;27(6):445–54. doi: 10.1136/bmjqs-2017-006784 (PMC5965349; doi:10.1136/bmjqs-2017-006784)

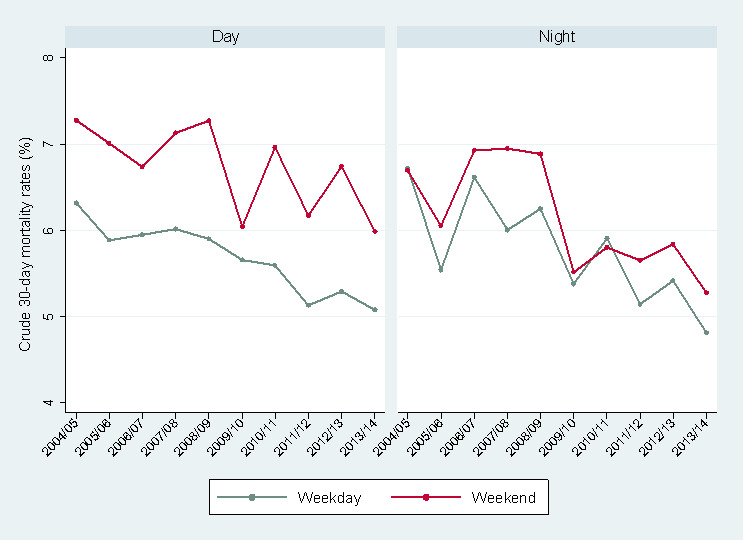

Supplement: Supplementary file 2 [file bmjqs-2017-006784supp002.jpg]
